# The Hospital. Nursing Section

**Published:** 1905-12-30

**Authors:** 


### The Hospital.

**Nursing Section.**

Contributions for "THE HOSPITAL," should be addressed to the EDITOR, "THE HOSPITAL" NURSING SECTION, 28 & 29 Southampton Street, Strand, London, W.C.

No. 1,005.—VOL. XXXIX. SATURDAY, DECEMBER 30, 1905.

### Notes on News from the Nursing World.

### THE UNITED STATES ARMY CORPS.

THE American nurses continue to show an extraordinary disinclination to add their names to the Roll of Honour. It is now nearly a year ago since the Superintendent of the Army Nurse Corps, Mrs. Kinney, issued an official intimation that it was desired to obtain the names of acceptable graduate nurses willing to serve in time of war or national emergency. This intimation, which obtained the widest possible publicity, and was followed by the issue of a circular letter by Mrs. Kinney to the superintendents of training schools asking for their co-operation in securing a representative body of women for the department of the public service, had the effect, in a period of six months, of inducing forty-one nurses out of a total estimated at upwards of 30,000 to join the Corps, the number being advanced by two at the end of seven months. Then, Miss Isabel McIsaac, one of the veteran nurses in the United States, to her infinite credit, asked that her name might be inscribed on the roll; and it was hoped that her patriotic example would have an effect upon the younger members of the profession. At the end of the year, however, Mrs. Kinney is under the necessity of stating that there are still only fifty-two names upon the volunteer list. This is all the more remarkable because, as Mrs. Kinney explains, the Surgeon-General only asks the graduate nurses to place their names upon a list and express their willingness to serve their country in time of need. As she affirms that "if, when the time comes for calling them out, a frail old mother or a bed-ridden father or other closer claims hold a nurse to her duty at home, she has only to say so, and there the matter ends," there is not the vestige of an excuse for failure to respond to the appeal. We cannot but think that in the coming year a sufficient number of American nurses will recognise the obligation of placing the Superintendent of the Army Nurse Corps in a position to signify that she has a sufficiently comprehensive volunteer list to provide for any emergency.

### THE LUNACY COMMISSIONERS AND GARTLOCH ASYLUM.

IN order to make the position at Gartloch Asylum quite clear, we have ascertained the hours of the nurses at the present time. They are on duty at 6.30 A.M. till 8 P.M., with three half-hours for meals. This makes a twelve-hours' day. One half-day from 2 P.M. every week is given as leave, and in every six Sundays one whole Sunday is given and a Sunday from 11 A.M. Once in six weeks the leave is so arranged that the nurses get away at 2 P.M. on Saturday and do not require to be back till 9 A.M. on Monday. This makes an average week of 75 hours, or if occasional theatrical entertainments and dances are calculated, 77.5 hours a week. These hours are as good as those of any asylum in Scotland, and better than the average. Moreover, as compared with other hospitals it should be remembered that the asylum patients are out walking regularly in the morning and afternoon with their nurses, and that even in the acute and sick wards open-air treatment in bed or verandah is the regular practice. The confinement is therefore mitigated, but even so the hours are exceedingly long. The Commissioners in Lunacy apparently think otherwise. They complained that Gartloch Asylum was over-staffed, the so-called over-staffing being due to the fact that longer leave was given in that institution than in any other asylum in Scotland. In consequence of this complaint, the Glasgow District Lunacy Board reverted to their former leave, hence the trouble that has arisen. It has been exaggerated, but we are not surprised that the nurses do not like the change. The onus, however, rests with the Commissioners in Lunacy, not with the authorities of the asylum.

### SAD SUICIDE OF A PROBATIONER.

A VERY distressing incident formed the subject of an inquest at Manchester last week. The inquiry was rendered necessary by the suicide of Miss Annie Armstrong, a young probationer at Crumpsall Infirmary who threw herself from a high window of the hospital on the previous Saturday, death being the result. Details have been published in the local papers, but we are able to supply some new and authoritative facts in connection with the case. Miss Armstrong only arrived at Crumpsall from Cork on November 27 in order to enter upon her training. She seemed in every way suitable and had good references. On December 13, another new probationer, Miss Annie Gibson, came from Belfast to Crumpsall and commenced her training. She had only been twenty-four hours in the hospital when she complained that a box containing her jewellery, a purse of money, and a new steel chain, had been taken out of a drawer in her bedroom. Inquiries were instituted without delay by the matron, Miss Girdlestone, and the result was that the matter was placed in the hands of the police, suspicions having fallen upon Miss Armstrong. Both nurses were interviewed by detectives, and on the morning afterwards Miss Armstrong committed suicide. It was stated at the inquest that money had been lost by the nurses on two or three occasions before the arrival of the deceased, but, of course, no attempt was made to clear up the question of her guilt or innocence. Since the inquest, however, all the missing articles, except a brooch discovered in the bedroom, have been found in a small cistern which supplies the lavatory. While we do not offer any opinion as to how far the discovery, and the circumstances preceding it, justify the suspicions which were entertained of the unfortunate girl, it establishes the fact that a theft was committed and that the culprit sought to get rid of the evidences of guilt.

### MIDWIFERY AND CHARACTER.

The importance of the utmost care being exercised by the clergy in giving certificates of good moral character to persons professing to practise as midwives cannot be exaggerated. There was a case in point mentioned at the last meeting of the Central Midwives Board, who are to be congratulated on the admirable manner in which they discharge the duty of dealing with persons reported to have been guilty of negligence or misconduct. In this instance they had to consider the action of a clergyman who had signed a certificate of good moral character for a woman within two months after her conviction for larceny and with apparent knowledge of her conviction. In reply to a communication from the Board asking for an explanation, he wrote that he "believed" her to be at the time of his signing of good character, and we have no doubt that he did. But in such circumstances it should not be a matter of belief; a certificate of good moral character should not be given without absolute knowledge. The Midwives Act was intended to raise the standard of midwifery, and has already had that result. It is, however, essential that in administering its provisions there should be the heartiest co-operation on the part of all responsible persons, especially in regard to the vital question of character.

### PROPOSED ROYAL COLLEGE FOR NURSES.

The current number of the *Westminster Review* contains an article called "The Nurse of the Future," by Dr. Oldfield. His principal contentions are that the size of the hospital in which the nurse has received her training is not of the high importance which it is assumed to be in some quarters; that there is no uniformity of training and no equalisation of certificate values; and that the public have no means of readily knowing who is and who is not a properly trained nurse. Dr. Oldfield's fantastic remedy is that there should be instituted a Royal College of Nurses, and that every nurse should be examined by the college, quite irrespective of any examination she may have passed at her own hospital, and that the diplomas of Bachelor of Nurses and Mistress of Nurses should be conferred on successful candidates, registration of such to follow on obtaining either or both of these diplomas. Dr. Oldfield has hard words for the uniforms now worn by so many nurses trained and untrained; nevertheless he suggests a special uniform for the proposed diplomates and any other nurse wearing it he would have adjudged guilty of fraud.

### SCOUTS FOR DISTRICT ASSOCIATIONS.

Why should not every District Nursing Association have its "scout" in the shape of a needlework guild? As to the value of organisations of this character it is only necessary to instance the results of the work done by the Guild at Cheltenham. This Guild has been in existence for eighteen years, and at the twenty-fifth annual meeting of the District Association in that town the other day Mrs. Brooke, one of the founders, was able to announce that there was every prospect of handing over "the usual sum" of £100 to the mother society, while nearly 2,000 garments had been contributed. The pecuniary assistance speaks for itself, and the value of the contribution in clothes can be readily conceived. Mrs. Brooke mentioned that the Superintendent of the nursing staff had told her that insufficient clothing for the bodies of the poor by day, and on their beds at night, was one of the main causes of eight pneumonia cases in one district; she also related the delight of a nurse who was assisting to open some belated parcels, when six very little hand-knitted vests tumbled out, and exclaimed, "Oh, won't these be lovely for my sick babies!" There are now 600 members of the Guild, and its success is entirely due to personal service in little matters. The "scout," as Mrs. Brooke happily called the organisation, thus not only provides more than the salary of a district nurse, but also renders material aid to the whole of the staff in their ministrations of mercy. We know, of course, that in Cheltenham there are numerous residents of private means and leisure; but what is done on a considerable scale there might be done in a smaller way in many towns with fewer well-to-do and more occupied people, while there are others in which results achieved at Cheltenham might be easily exceeded. In fact, there are few districts in which effective assistance in a greater or less degree could not be rendered. We hope to hear that the spring of 1906 has been marked, in many instances, by starting the scout who has performed its mission so admirably in Cheltenham.

### AN INSPIRING VISION.

The President of St. Catherine's Hospital, at Cawnpore, Miss Macnaghten, in a paper read at a conference of nursing superintendents of the United Provinces and the Punjab, said that she had visions of one day seeing a little group of Indian lady nurses discussing the work of improving the standard of nursing. Miss Macnaghten believes that the nursing of Indians could be best done by natives, and we are satisfied that her ideal is the one to be aimed at. But there is a good deal to be done before it can be fulfilled, and it is a matter for congratulation that meanwhile the number of trained European nurses in India is steadily increasing. It is for them to use their opportunities in such a manner as to stimulate suitable native ladies to qualify themselves for the work.

### OATHS OF SECRECY.

It has been suggested that as a remedy for gossip on the part of nurses, the authorities of training schools in this country should follow the course pursued in New York, where forty-one nurses who have just received their certificates took an oath not to divulge the secrets of their patients. We are afraid that a certain number of nurses allow their tongues to wag too freely, and to discuss matters that should not be mentioned outside the four walls of the sick-room. We say the sick-room because it is the private nurse rather than the nurse in the ward of a hospital or an infirmary who is more tempted to retail gossip, because she is more likely to be supplied with material. Those who indulge in this most reprehensible practice do not always realise that they may cause wide-spread mischief, and that they are actually guilty of a serious breach of trust. But we do not believe in oaths of secrecy as a remedy for tittle-tattle. For the nurse who is discreet and who understands the obligations imposed upon her, an oath is superfluous; and for one who is indiscreet and has no natural or acquired sense of the importance of not betraying the trust necessarily imposed in her, it is a mere form which is as likely to be broken as to be observed.

### THE RISKS OF ELECTRIC RADIATORS.

A report just issued to the managers of the Poplar and Stepney Sick Asylum through their Visiting Committee shows that the Administrative Block of the building narrowly escaped destruction on the 25th of last month. The Medical Superintendent states that a fire occurred on that day in a nurse's bedroom in the block, which, on inquiry, was proved to have originated by the ignition of clothing hung out to air coming accidentally into contact with an electric radiator. In consequence of the presence of mind and fearless conduct of the nurses who discovered the fire, the Medical Superintendent thinks that a serious outbreak was checked with the loss of only a chair, and a wardrobe slightly scorched; and he has suggested that some other means of heating the room should be adopted in place of the electric radiator. The managers have not acted upon the suggestion, but have instructed the clerk to issue a note of warning to the nurses as to the danger of putting the clothes near the radiators to air. We hope that this will be sufficient to prevent mishaps in the future. An electric radiator is not the most efficient nor the most economical means of heating a room, but, with ordinary care, it is not a source of peril.

### CROYDON POOR-LAW INFIRMARY.

There are two interesting items of intelligence from Croydon Poor-law Infirmary. One is that the Croydon Guardians have approved a report of the Infirmary Committee recommending that the salary of the matron should be increased from £100 to £120 per annum. The other decision at which the Guardians have arrived is to drop the outdoor nursing scheme. This is owing to the fact that the Special Out-Relief Committee, in the course of their investigations, only found two cases which might be nursed outside the Workhouse Infirmary. A proposal to refer the matter back was lost, which was just as well. We think it is preferable that the whole of the District Nursing should be done by a staff quite independent of the Poor-law authorities.

### NURSING ERYSIPELAS IN INFIRM WARDS.

A male nurse in a workhouse, which he does not name, sends a letter to a contemporary in which he states that three months after his appointment twelve months since to the charge of aged and infirm men a case of erysipelas was sent to his ward and that from that date forward he has invariably had one or two cases. At present he has four; and in addition, he now has to nurse patients suffering from measles and chicken-pox in the same ward. The nursing of infectious cases, he adds, is not mentioned in his appointment, but he has been told, in effect, that if he does not like the work he can leave it. We fear that in a case like this no remedy is possible without the name of the institution being given. To treat erysipelas, or any other infectious disease, in an infirm ward is, of course, contrary to the regulations of the central authority, and if the attention of the Local Government Board were drawn to this gross breach of duty, there is no doubt that effective action would be taken.

### QUEEN'S INSPECTORS.

We announce to-day the appointment of another new inspector under Queen Victoria Jubilee Institute for nurses, in the person of Miss Glover, who since November 1898 has held the post of superintendent of the Lincolnshire Nursing Association. Two more of these important appointments, which offer great scope for a capable nurse, are still open. The preference will be given to a Queen's nurse who holds the certificate of the Central Midwives Board and is a cyclist. The salary offered is £85 a year with furnished rooms and attendance. Application should be sent in before January 6 to the Secretary of the Queen Victoria Jubilee Institute, 120 Victoria Street, S.W.

### THE ROYAL MATERNITY CHARITY OF LONDON.

In the year 1906 that excellent institution, the Royal Maternity Charity of London, will celebrate the 150th anniversary of its institution, and at the annual general meeting of the governors on February 20, which will be held at the Mansion House, the Lord Mayor in the chair, the most suitable mode of commemorating the event will be discussed. All well-wishers of the charity will be welcomed at the meeting and cards of invitation will be sent on application to the Secretary, Major G. Lionel B. Killick, 31 Finsbury Square, E.C.

### SHORT ITEMS.

The Ecclesall Bierlow Guardians have decided to subscribe £200, with £50 for necessaries, to Queen Victoria Jubilee Institute for Nurses.—An influential committee has been formed for the purpose of obtaining a district nurse to work the parishes of Horrabridge, Meavy, Sheepstow, and Sampford Spiney. The association will be affiliated to the Devon District Nursing Association, and it has been decided to invite Sir Massey Lopes to become first president.

### The Nursing Outlook.

> "From magnanimity, all fear above;
> From nobler recompense, above applause,
> Which owes to man's short outlook all its charm."

### PAST AND FUTURE.

The year 1905, so far as nursing matters are concerned, will be memorable because it witnessed the bringing together of all shades of opinion in the United States. In England public opinion has been quickened in regard to nursing affairs, and the growth in knowledge and sympathy amongst all classes has been marked. All who have the interests of nurses and nursing work at heart, who are striving so earnestly for their improvement, as Miss Monk has well said, desire some unanimous decision that would once and for all place nurses in their true position, and guard them from all future misconception and disrepute. Such a decision should restore the nurse and her work in the eyes of the world to the noble position they held when the refined and beloved Florence Nightingale was still strong enough to be their leader, when nurses were held to be the pick of their sex, and were supposed to be above feminine jealousies and party politics. We have often said that misconception and disrepute have too long dogged the footsteps of the modern nurse in Great Britain. The only effective remedy is to set up a central authority, with the cooperation of some of the more intelligent and public-spirited nurse training schools and teachers and instructors of probationers, and to constitute it by charter or incorporation on an authoritative basis. We hope that such a Central Council of Nursing Education may be established during 1906, and that it will throw its examinations open to all nurses wherever trained, decide upon a definite course of consecutive training, and issue a standard certificate representing the average requirements. Such a certificate should include evidence as to the moral and personal qualities of each nurse, certified by those who have had her in charge during her period of probation. An organisation of this kind must originate in this country by voluntary effort. It will pave the way to Parliamentary action, which cannot be effectively undertaken until the decks have been cleared by sifting out the really competent among the tens of thousands of nurses who have not had the advantage of a three years' hospital course, though they are working in most divisions of the nursing field. The House of Commons Committee on Registration did good service by collecting some useful evidence on the subject, which confirms the accuracy of this view.

The Midwives Board has settled down to steady, useful work, and although an amending Act will no doubt be necessary in a few years' time, the Board has justified its existence and is advancing in favour. Its proceedings show the wisdom of having women representatives upon a Board of this character, for the discussions have proved that they understand the question in all its bearings, and have thus saved the Board from making many serious mistakes. The Queen's Jubilee Nurses' Institute has lost the services of Miss Peter, under whose guidance it has extended its work and increased its usefulness, to a marked degree. Miss Peter was presented with a notable testimonial, which she had well earned, and everybody will wish her much happiness and good health in her retirement. Her successor, Miss Hughes, is deservedly popular in the nursing world. Her powers of organisation are considerable, and her energy and influence in district nursing matters place her at the head of this branch of her profession. Two matrons who have done good service have retired from office during the year. Miss Thorold, who has held the office of matron to the Middlesex Hospital for 35 years, is widely known and esteemed, and her presence will be missed from several fields of work where her influence has had a material effect in shaping the policy and providing initiative. Miss Wedgwood, whose services at the Royal Free Hospital have been gratefully recognised, had to relinquish her work for private reasons at a time when her influence was extending, and her counsel was being increasingly sought by her contemporaries. It is not possible to record all the other changes of the year, which have duly been recorded in these columns, but we wish all who have retired from office after faithful service many years of enjoyable leisure, and those who have entered upon greater responsibilities the judgment and character to enable them to make their mark and so advance the interests not only of the immediate work entrusted to them but of the whole nursing profession.

Most people are in the habit of taking stock at the commencement of a new year, and the majority have the wisdom to look forward rather than backward. The older one grows, the more one realises the wisdom of striving to rise on our dead selves to higher things. It is so true, that the world regards the position of each man or woman from the point of view of the work which has been successfully accomplished, whilst they themselves are animated by the feeling of the power they have within them to do and dare in the cause to which they have set their hands. Cheerfulness in the sick is the high road to recovery, and cheerfulness in the healthy promotes progress and encourages hope. A lady of mature years and keen intelligence, who belonged to a past generation, after health regarded hope as the first essential to success. We cannot do better than print her couplet for the encouragement of our readers at the commencement of 1906. Here it is:—

> Begin the year with increased hope,
> For hope is energy begun,
> And energy success.

### Some Essentials in the Nursing of Infectious Diseases.

By A. Knyvett Gordon, M.B.Cantab.; Medical Superintendent of Monsall Hospital; Lecturer on Infectious Diseases in the University of Manchester.

(*Concluded from p.* 168.)

In my last article I touched on some of the requirements for the successful nursing of infectious disease, and I propose now to describe briefly how these essentials may be carried out in practice.

The first point that I wish to make is, that not only is any one disease in a fever hospital "catching," as we call it, to a patient suffering from any other, but the illness from which a patient is suffering may be, and often is, conveyed to another child suffering from the same disease. Let me illustrate what I mean by this. It is quite apparent that, if into a scarlet fever ward a child who has, not scarlet fever, but diphtheria, is admitted, he has a very good chance not only of catching scarlet fever himself, but of giving diphtheria to the other patients.

But it may not be equally evident that a patient who has scarlet fever may be infectious to other scarlatinal patients. You may say, "Oh! they have all got the same disease, so how can I carry infection from one to another?" Yet you can, and I am sorry to say you do. The fact is that we very seldom have, in any disease, what bacteriologists call a "pure infection." If we examine the throats of patients suffering from scarlet fever, for instance, by taking swabs from the tonsils, we find, when the germs that have grown in the incubator in the laboratory from these swabs are investigated, that we have, not one germ, but many different varieties of organism. And the assortment is scarcely ever the same in any two patients. So much for theory; but what happens in practice? Well, I have seen a collection of children in one scarlet fever ward who were all going on nicely towards convalescence; their throats were healing and their temperatures normal. Then there was admitted into that ward a boy with a very severe attack of scarlet fever, whose throat was extensively affected. Shortly afterwards one child after another had a rise of temperature, followed by ulceration of the throat; in fact, to use a common and very expressive term, this throat "went round the ward." This occurred, I may say, in a ward where the nursing was far from satisfactory.

The next thing to remember is that infection of "infectious" diseases is not carried entirely by the air. I believe it seldom travels far in the air, unless gross overcrowding exists in the ward. Infection is conveyed in "medical" cases just as it is in "surgical" ailments, by unclean hands and clothing. If, in a general hospital, the wound, after the operation for a radical cure of hernia, for instance, suppurates, the surgeon will think first of his own hands, and of the sponges, ligatures, etc., and then perhaps of the hands of the nurses; but he will not talk about infection from the air very much. And it is just the same in fever cases: if a child develops another ailment, or a different variety of the "same" disease, the careful nurse will think of her own hands, and of the patient's clothing, feeding utensils, etc.

To realise these two points is not only to diminish infection amongst the patients, but to take away from "fever nursing" the irksomeness which is inevitable as long as it is regarded merely as a struggle to obey a set of apparently meaningless rules, or to escape detection if you do evade them.

So what we have to aim at is clean hands and surroundings. Now, it is all very well to talk about the necessity for thoroughly sterilising the hands before doing anything for a patient in the way of treatment, but when we think that to perform this operation at all satisfactorily by any method takes at least ten minutes, and even then the hands are frequently not quite sterile, though they may be visibly very clean, it will be seen that when even six patients have to be treated, the toilet of the hands takes an hour. It is obvious that this process is more often talked about than adopted, and, incidentally, this constant repetition of the sterilising would very soon make the hands rough and chapped. So we must find some other method. At Monsall Hospital, where the throat, nose, and ears of every patient are cleansed at least once, and often more frequently, during the twenty-four hours, and where a ward contains at least twenty patients, the method adopted is as follows:

A travelling dressing-wagon is provided carrying two sets of vessels, for clean and dirty articles respectively, the latter being distinguished by their containing a small quantity of a 1 in 200 solution of izal fluid. Before starting on her round the nurse sterilises her hands by scrubbing them with pure soft soap (prepared on the premises from pure sterilised fat) and a nail brush; this is followed by rubbing with turpentine, and then with methylated spirit. The hands are then covered with recently boiled rubber gloves, which are kept on until the round is finished, the gloved hands being cleansed between the treatment of each patient by holding them under the cold water tap for two minutes, followed by immersion in a solution of biniodide of mercury, or of izal 1 in 200. By this method all danger of infection from hands is eliminated, and the hands themselves are rendered softer, not hard and chapped, by the wearing of the gloves. Moreover, the risk of "septic fingers" on the part of the nurse is practically eliminated. Then the throats are irrigated with a douche, not syringed, it being quite impossible to prevent infective matter from the throat escaping into the body of a "Higginson's" or of a rubber-ball syringe. With a douche no such infection of the instrument can occur. For each patient a separate recently boiled bone nozzle is used, and any swabbing of the throat is done with sterilised swabs, which are taken for the purpose out of boracic lotion.

Infection, however, does not come only from hands and instruments. It may arise from the inhalation of germ-laden dust.

Now the remedy for this is "menial" work; in other words, constant scrubbing of all cupboards, shelves, etc., with damp dusters, and, most important of all, sweeping of the (polished wood) floors with brooms covered with *wet cloths*. Now, when it is realised what this means to the patients, it will be obvious that it must be done by the nurses, to whom it should mean something, and not by the ward maids, to whom it can mean nothing but extra labour. And while on this subject, I wonder whether a junior nurse, amidst the novelty of the "menial" work, and the grumbling it entails, ever thinks that the experience may be a valuable asset to her later on, if she remains in hospital work and becomes a sister or a matron? She will then be responsible for seeing that this part of the work is done properly. How can she do so if she has not done it herself?

The ways in which infection may be conveyed by the surroundings of a patient are many. Here, again, once the habit of asepsis is attained, the nurse will know by instinct what is capable of conveying infection and what is harmless. Perhaps the most important source of infection, however, is the secretion from the nose, mouth, or from a discharging ear. These secretions may get on to pocket handkerchiefs, towels, pillow-cases, etc., and from them be easily conveyed to another patient. Toys that are put into the mouth are a fruitful source of offence in this way also. Except in the case of discharging ears, where the secretion can be prevented from escaping to the child's surroundings by covering them with cotton wool and a bandage, it is almost impossible to prevent linen, etc., becoming infected, but it is quite possible to prevent any contact between the linen and another patient. Thus it is not right for a number of convalescent children to wash their faces at the same time, and then dry them on the same roller towel; nor is it right for a patient with nasal discharge to carry a pocket handkerchief at all. He should have small squares of cheap muslin, or of Japanese paper, which can be burnt after use.

Examples of the necessity for precaution in the nursing of infectious disease might thus be multiplied almost indefinitely, and the details are best learnt in connection with each particular disease. When these differ so widely in their nature, as, for instance, puerperal fever and scarlet fever, or erysipelas and diphtheria, it will be seen that the whole art cannot be learnt in a day, or even in six months, but the principle is the same—asepsis everywhere.

### The Nurses' Clinic.

### INFANT FEEDING.

Every nurse knows that the ideal food for a baby is the mother's milk, and further, that he should have nothing else till about eight months old. This is perfectly true, but unfortunately one so seldom attains the ideal, and in so many cases have to substitute the next best. The mother may be willing and anxious to nurse her child, but she may be delicate, anæmic, tubercular, or epileptic, or it may be that her milk is poor in quality or quantity, or even absent altogether. Any one of these causes are against her feeding the child, both for her sake and his.

It is a great mistake to persist in breast feeding a baby if he obviously does not thrive, does not gain weight, or sleep well. If the mother's milk does not satisfy some substitute must be found, either a healthy wet nurse or hand feeding. The choice of a good wet nurse is difficult and anxious, so much so that the mother often prefers the latter alternative, and with good cow's milk within easy reach it is perhaps the wisest course. There are three natural substitutes for human milk—namely, ass's milk, goat's milk, cow's milk. The first is expensive and not always obtainable, the second is sometimes to be had when cow's milk is scarce, as in Malta or abroad; the third is the best and cheapest and the one most commonly employed.

Cow's milk differs in its constituency from human milk, as the following table shows :—

| *Woman's milk.* Average. | | *Cow's Milk.* Average | |
|---|---|---|---|
| Fat | 4.00 | Fat | 3.50 |
| Sugar | 7.00 | Sugar | 4.30 |
| Proteids | 1.50 | Proteids | 4.00 |
| Salts | 0.20 | Salts | 0.70 |
| Water | 87.30 | Water | 87.00 |

To render it equal to woman's milk it will be necessary to (*a*) Reduce the proteids by adding water. The addition of water tends to prevent the formation of the hard, indigestible masses which undiluted cow's milk produces in the stomach. Human milk produces loose, flocculent curds. (*b*) To increase the fat by adding cream. (*c*) To increase the sugar by adding sugar of milk.

To make this the ideal substitute for mother's milk the milk *must* be fresh and sweet, and from healthy animals, preferably from a mixed herd than from one cow, as the latter is more subject to daily variation.

If this mixture suits the baby he will gain weight, sleep well, his flesh will be firm to the touch, his colour good, and his stools will be normal, regular, and healthy. Should they be green, or hard, or fluid and frequent, something is wrong, and that something is usually the food or in the feeding. He may need his milk more diluted or less food at a time, or perhaps the bottle was not perfectly clean (rubber teats have to be most carefully overhauled), perhaps he took it too greedily, perhaps the temperature of the food was not right.

Hand-fed babies are as a rule more often overfed than underfed, and when that is the case they usually retort by vomiting. I have heard some mothers say "they would not give a ha'porth for a baby who did not throw up his food; it was such a sign of health." The trained nurse, however, would reduce the amount of food at a meal, to the benefit of all concerned.

With infants it is important that they should be fed at regular intervals, that the food should be freshly prepared each time, not warmed up. The bottle must be held in such a manner that he does not take it too fast or suck in as much air as milk.

The stools must be examined daily for signs of undigested milk, etc. The following schedule by Dr. Louis Starr may be useful :—

| Age. | Average amount at each feed. | Interval between food. | Average amount in 24 hours. |
|---|---|---|---|
| First week ... ... | 1℥ | 2 hours | 10℥ |
| First to sixth week... | 1½ to 2℥ | 2½ hours | 12 to 16℥ |
| Sixth to twelfth week and possibly to five or six months ... | 3 to 4℥ | 3 hours | 18 to 24℥ |
| At six months ... | 6℥ | 3 hours | 36℥ |
| At ten months ... | 8℥ | 3 hours | 40℥ |

If an infant's digestion is very feeble, as in the case perhaps of a premature baby, the doctor may think fit to order the milk to be peptonised. Fairchild's peptogenic milk is useful under such circumstances, but as with adults, pre-digested milk is only regarded as an emergency measure, and reduced as soon as possible. Cheadle looks upon peptonised milk as "a valuable temporary food," but "it weakens the digestive powers, and he has seen more than one case of scurvy result from its use."

After the teeth have begun to appear and the salivary glands are developed some one or other of the well-known patent foods may be given once or twice a day. It is not advisable if the child is thriving to try them before he is six months old, as, though the pictorial advertisements of large, plump babies "fed from birth entirely on So-and-So's food" are very alluring, nearly all patent foods contain starch in a more or less degree, and the stomach has no power of digesting starch for the first three months. Neither in proprietary foods is there the same quantity of fat and proteids so necessary to growing organism.

Sometimes, however, patent foods succeed where others fail. Paget's milk was successful in rearing a feeble, premature infant who relapsed directly he was given ordinary milk and barley water. It sometimes happens that milk in any form disagrees; in that case an ounce and a half of chicken broth or whey, or half an ounce of raw beef juice will be retained and tide over a few days till milk food can be resumed. Most infants digest cream well, and Ashby and Wright recommend a trial of four ounces of cream to twelve ounces of warm water, with half an ounce of milk sugar when cow's milk cannot be digested.

### Incidents in a Nurse's Life.

### "OUR CHRISTMAS PARTY."

It is the night of our annual party in Ward VI. of a big Children's Hospital. Sister H. always gives one, and invites all who can come, both patients and nurses, from the other wards.

Ward VI. looks lovely; draped from the ceiling on invisible wires hang festoons of pale pink muslin, interspersed with Chinese lanterns and big white birds, which apparently are floating in mid air. The white tiled walls are hung with long trails of ivy, and every available bracket holds pots of ferns, while the table itself is a mass of lovely flowers and fronds, among which gleam coloured fairy lamps and glittering tinsel. It is a veritable fairyland to the little occupants of the cots, who have followed the decorating with keen interest.

"Do dress us now," pipes a wheezy little voice from one cot.

"All right, Tommy; one moment," answers nurse cheerily, pushing cots nearer together and arranging borrowed tables and chairs for the expected guests. That work finished, she and her "pro" prepare to dress the little patients; not that their toilet is a long process—it only consists in putting on new scarlet flannel bed jackets and big frilly white collars, and the small girls have scarlet hair ribbons and the boys smoothly brushed pates.

Before they are quite ready "Sister" arrives to give the finishing touches and set out the tea, and then it is 4 o'clock, and our guests are always punctual.

"May we come in, Sister," says a gay voice at the door, and Nurse G. from Number III., a surgical ward, has arrived with her contingent, she and her pro. both carrying a "hip case," and followed by half-a-dozen bandaged or crutched little followers.

"Is that all, nurse?" asks Sister.

"No; I've got to go back and fetch Owen, the 'appendicitis,' who has been in so long. Mr.—— said this morning he might come. He was so anxious to, poor little chap."

"That's right; he shall go into the empty cot," says Sister, and the other little visitors are soon accommodated with chairs or couches.

The guests are arriving in good earnest now, and soon every place is filled, and tea is only waiting for Matron and the Chaplain.

But they have not long to wait, and soon all the good things are disappearing with startling rapidity.

"Well, old chap, enjoying yourself?" asks the house surgeon as he offers a plate of sponge cakes to a small fellow, whose scarlet coat has a large ticket pinned to it bearing the legend "No Jam."

"It's proime, sir," is the heartfelt answer, the little white face aglow with enjoyment.

Nurses and doctors are hurrying about with good-natured jests and laughter waiting on the happy, expectant children, and everyone is as gay and merry as possible, as if pain and suffering were unknown, though the thin little cheeks and wasted frames tell another story. Even little Elsie, a "heart case," on whose account "Sister" had almost feared she would have to give up the party, sits propped up in her cot, too tired of this wearisome life to talk much certainly, but with a wan little smile on her lips and a half-eaten cake in her hand, enjoying herself as much as any.

"Well, dearie," nurse says, "I wonder if Father Christmas is going to bring you the dollie you want so much; I expect so," and she bends tenderly over the languid little figure, thinking how very short a time this little suffering human atom will be here to want anything.

"Ain't my 'air ribbin nice," the mite whispers. "It's all my own," with pathetic vanity, even under the grim shadow of the Dark Angel.

"It's lovely, dear," nurse says kindly.

"Do you think 'eavin is as pretty as this, nuss," she goes on. "Mover said last visitin' day I was goin' to 'eavin, and I'm orful tired."

"Heaven is much, much prettier than this," nurse's kind voice assures her, "and there is a Great Good Doctor there Who will make you quite strong and well, and Who loves all the poor little sick children."

The blue eyes gaze up into the face above them with infinite faith in their depths. "I shall ask 'im to come and fetch my Willie too then—my Willie wot 'as a bad back, yer know, nuss," and the little voice trails away into silence again, though the little brain is thinking of various requests to make of that kind Doctor when she sees Him, for the suffering that has been around her all her short life.

Nurse has to leave her now, for tea is over, and the great festivity of the evening is about to commence.

Perhaps few had noticed that the house physician had disappeared about half way through tea, and now the door opens and an old old white-bearded man, in a scarlet cloak and bearing a huge sack, appears, though the very modern looking boots and legs below the scarlet cloak tell the observant who he is.

But faith is such a huge factor in a child's existence, and a cry of "Father Christmas! Father Christmas!" echoes through the ward.

"A merry Christmas, children," he says in a deep voice, and then, reaching the centre of the ward, he stops and opens his sack. There is a toy for everyone; beautiful dolls for the girls and horses or mechanical toys for the boys, and at the bottom of the sack heaps and heaps of crackers. Oh what a noise goes on! exclamations of delight as the waxen babies or motor-cars, etc., are examined and discussed, and then the banging of crackers, many of which contain ear-piercing whistles, which add to the general din. At last the Chaplain stands up. "One moment, children," he says, "don't you think we ought to thank Sister H. and the nurses for this lovely party, and Father Christmas for the beautiful toys? Come along now, altogether, Hip, Hip, Hurray!" And wild cheers break out, making quite a respectable noise, in spite of some of the weak little voices.

"Sister" stops them at last, with a merry "That's enough, children, thank you; now I'm afraid it's good night." And the various guests are helped or carried back to their respective wards, full of the evening's delights, and longing to recount them to those unlucky ones who were too ill to come, but whom, they discover, to their surprise, Father Christmas has not forgotten.

### To Nurses.

We invite contributions from any of our readers, and shall be glad to pay for "Notes on News from the Nursing World," "Incidents in a Nurse's Life," or for articles describing nursing experiences at home or abroad dealing with any nursing question from an original point of view, according to length. The minimum payment is 5s. Contributions on topical subjects are specially welcome. Notices of appointments, letters, entertainments, presentations, and deaths are not paid for, but we are always glad to receive them. All rejected manuscripts are returned in due course, and all payments for manuscripts used are made as early as possible after the beginning of each quarter.

### Christmas in the Hospitals.

BY OUR OWN COMMISSIONERS.

If, as our Christmas Number testified, the Christmas season is now observed with spirit in the mental asylums whose inmates suffer from disabilities of a different character from the patients in general hospitals and infirmaries, this must be regarded as yet another result of the practice which had its origin in voluntary institutions. The practice has, in fact, become usual in all representative buildings where the sick or infirm in body or mind are detained; and the most pessimistic cannot but admit that here, at any rate, is a development which admits only of congratulations, so long as the underlying motive is the same that prompts the charitable to contribute money for the purpose of affording our poorer fellow-creatures, in their hour of sickness and distress, the fullest advantages obtainable from medical skill and trained nursing. No doubt there are contributors to Christmas entertainments in hospital who are not without a sense of self-importance or a desire for self-glorification. But these, we are quite convinced from long observation and experience, are a very small, not to say fractional, minority. The raw probationer, or the newly fledged medical student, who may at the outset derive satisfaction from any compliments that may be paid to a natural taste in decoration, or a natural aptitude for affording amusement, soon has the taint of egotism removed by the heartfelt enjoyment derived from the contemplation of the gratification of others. The primary object of all the simpler arrangements and all the more elaborately planned programmes, of which many details are supplied by our Commissioners, is, of course, to ameliorate the lots of those who have to spend Christmas in hospital wards instead of in the home circle; but the gain to those who give the pleasure, by the cultivation of the higher form of unselfishness which is brought into play on such occasions, is very real, and tends to render the seasonable entertainments that give rise to so much genuine happiness a means of strengthening the best side of character.

### Guy's.

Christmas Day at Guy's kept up its reputation to the letter. There was brightness, music, and gladness everywhere. Even the worst patients seemed cheered, and the better for the surrounding gaiety, and the convalescents could not say enough in praise of the "wonderful arrangements" for their comfort and pleasure. The wards were decorated with evergreens and crinkled paper of delicate colours. Each ward carried out some particular scheme of colour and design. Lazarus ward was a dream of loveliness in mauve. Clusters of lighted paper bells being suspended at intervals, it richly deserved its epithet of "la belle" ward. Astley Cooper ward was to the front with originality; two windmills, one each side of the entrance door, decked with Guy's colours—orange and purple—were the special features; being covered with art muslin and lighted inside with electric lights, the effect was charming and unique. Every ward was so tastefully and prettily decorated that it is difficult to know which should receive the palm. The nigger troupe—composed, as usual, entirely of residents, dressers, and ward clerks—was a complete success. The grotesque costumes, and the more grotesque antics, and the funny songs and jokes were deservedly appreciated by everybody. "The niggers" were never short of an audience, for many visitors were present in every ward. The patients, of course, being placed well to the front, had the best and first chance of seeing and hearing. One old daddy remarked, at the close of their performances: "Well, I never thought they was all doctors; wonderful good of 'em to take all that trouble!" In the out-patient department some hundreds of poor children had tea and a gift from the beautiful Christmas tree. The rooms for the visitors in this department were works of art. Paper flowers attached to boughs formed a complete roof in one room, while the festoons of Japanese appearance made beautiful another room. The large rooms for the children were hung with bunting and Chinese lanterns. It is worth mentioning that the front surgery was especially quiet, casualties being few and of minor importance. It was gaily decorated with evergreens, flags, and Chinese lanterns. Everybody agreed at the end of the day that it had been a most happy and successful time, and as the lights went out, and the presents which every patient had received were put away, one felt that many patients were thankful to have been able to enjoy a hospital Christmas. During the week various concerts are going on, and the residents are giving theatrical performances three evenings for the amusement of the nursing staff and some visitors. The week of festivity ends with the old custom of the residents dancing round the statue of Thomas Guy by the light of a bonfire on New Year's eve.

### St. Bartholomew's.

Under no circumstances do the massive grey walls and stately porticos of St. Bartholomew's Hospital look better than when night has fallen on the historic quadrangle, and from the rows of windows surrounding it streams the light from the brightly illuminated wards with their ruddy leaping fires. The sight has a chance to grow monotonous these short December days; but on Christmas Day the Square had lost its usual hush towards the end of the day, and from four to six it was gay with the laughter and hurrying feet of doctors, sisters, nurses, and visitors flitting from ward to ward in the different blocks to see the decorations and entertainments. Sisters and staff nurses took it in turn to leave their own wards and "do the round," though some, alas! were so busy that they were obliged to content themselves with their own particular triumphs and verbal accounts from the juniors of what was going on elsewhere. Pitcairn, for instance, which was "taking in," had received eight fresh cases in two hours, seven of them being broken legs. The hospital was full, and in the tiny two-bedded wards leading off the big surgery were two wee babies very, very ill; and perhaps the parents, watching in grief and anxiety over the frail little lives, were glad that the surgery, in its quiet corner by the Chapel, is some distance from the wards where all was gaiety and enjoyment. As usual, students and nurses had laid themselves out to give an entirely happy day to those to whom they minister. Off-duty hours had been spent for some days in making ropes of evergreens, fancy decorations, and presents. All the electric lights were covered with scarlet shades, which shed a warm glow over the white beds and shining brass and pewter. The women patients found a renewed interest in life in dressing their hair and arranging their bows and ribbons to the best advantage. In Paget the children were all dressed in blue frocks of nun's veiling, with white pinafores, and blue ribbons in their hair, and sashes to match; so they looked like part of the decorations themselves. There was a profusion of beautiful flowers, and fairy lamps were dotted about all over the place—suspended from the festoons of evergreens, perched on the medicine brackets over the patients' beds, nestling among the ferns and palms banked up on the marble slab at the end of the ward. Tea was going merrily in all the wards, and there was a plentiful supply of cakes and fruit and sweetmeats for everyone. Pulling crackers is a favourite

CHRISTMAS IN THE HOSPITALS—*continued.*

treat, and the gaudy paper headgear and other apparel hastily donned by the patients all added to the fun. Several of the wards had a Christmas-tree, laden with pretty gifts for old and young, and a great feature of Paget, which caused much mirth, was an array of Dutch dolls which sister had produced from some old store of treasures. The students had got up a nigger performance, which they gave in most of the wards in succession. They were followed by a conjuror, a gramophone, and an excellent baritone, who were all engaged by the hospital from outside. The singer was accompanied by a troop of dressers, who carried the piano about from ward to ward. The hearts of his audience were stirred to enthusiasm by such songs as "Sweet and Twenty," "Devon, Glorious Devon," and the children were only just restrained from joining in the accompaniment with their new trumpets and drums. Recitations naturally formed part of the entertainment. Mark Ward had a little private play and concert of its own, given by some friends of the staff, and a real printed programme was issued! "Leave It to Me" was the title of the "Farce in One Act. By Arthur Williams," and the players showed themselves equally proficient in the dramatic and the musical arts the instrumental quartets and violin solos being much appreciated. But sick people cannot stand unlimited excitement, and at six o'clock the sisters began to close their wards, and the great day was at an end.

### Middlesex.

A series of entertainments, simple and as homelike as they could be made, has been going on this week at the Middlesex Hospital, all provided by the hospital staff or its friends. These have been hard at work for a long time, and the fund out of which the decorations, presents, cakes, etc., were bought was collected by them. The Matron, to whom fell the task of purchasing nearly everything, has been indefatigable in her efforts, and has ransacked the toyshops of London, buying motor-cars, dolls, trains, furry animals, and boxes of chocolate by the gross wherewith to deck the Christmas-trees. The day began at 5.30 a.m., when the Chaplain, who has not spared himself either, held a service (Holy Communion) for the servants. Services followed at 6.30, 7.30, and 9 for the nurses, sisters, and other officials, in order that all might have an opportunity of coming; and at 10 there were Matins, as usual, a few of the patients also attending. All the work of preparation and decoration had been done by the nurses and servants, the students rendering useful assistance in dressing the Christmas-trees, which are prickly subjects to handle. Different schemes of colour were carried out in the various wards in lamp-shades and flowers, and there were some lovely combinations of scarlet and green, yellow and white, green and yellow, and orange tones. Christmas fare was indulged in by all to whom it could possibly be allowed, and the change was a highly welcome one. Several turkeys were presented to the hospital, and some of the visiting physicians and surgeons sent them specially to their own particular wards. Not only did they give the turkeys, but they came and carved them themselves in the wards, with a nice appreciation of the cooking and a careful consideration of the fitness of the joints which they apportioned to each of their patients. Then came the visiting hours, and from two to four the patients' friends arrived in extra large numbers to give and receive the familiar greeting. Sunday, Tuesday, and Friday are the ordinary visiting days, and Monday was an "extra" thrown in, so the patients have had quite a run of "society." At four o'clock they left, and the friends of the nurses, doctors, and students began to flock in. A plentiful but dainty and delicious tea was served in each of the wards, the nurses and servants being allowed to circulate all around the hospital, but entertaining their own visitors in their own wards. There was a present for everyone; not even the green parrot (a great pet with patients and nurses) in the Cancer Wing was forgotten. The nurses were charmed with their pretty cups and saucers, and the patients in the women's wards could not resist putting their pretty shawls or crossovers on over the new red jacket in which each was attired. A packet of tea and a cake of soap ("Such *nice* soap!" one woman confided to me) were distributed all round, and the men had shirts instead of shawls. In the Cancer Wing each of the thirty-six women had been presented with a pretty soft white wrap by Lady Ardwicke, made by herself. Here writing-cases and scent or smelling-salts were substituted for the tea and soap which the cancer patients do not provide for themselves. Then there was a huge lucky tub carried round—three dips for everyone. The Chaplain gave a gramophone entertainment, and the nurses in some of the wards sang carols. But perhaps the children's wards were the most attractive of all. In the centre of Princess May the babies' ward stood a giant Christmas-tree, its boughs bending under the weight of hundreds of delightful toys, with many electric lamps ingeniously carried up into the foliage. Never was there such a tree or such a fairy sight for little sick folk to gaze upon! Dolls, big and little, dark and fair, of every rank in life to judge by their dresses, sat or lolled on the chairs, lockers, and window-ledges, and each cot-tray had its own collection of toys—the precious possession of the tiny sufferers who, in some cases, could only lie and gaze at them with big, wondering eyes. It is pleasant to think how much unselfish thought and loving generosity of other children, strong and lusty in their happy homes, those toys represented. There was another tree in Percy, where the boys range from two to eighteen years, and here the toys were more suited to babies of a larger growth. Two little lads—one with a helmet and the other a sword—were marching up and down, playing at soldiers. Poor little wights! They had very surely been in the wars, for one had lost a foot.

### Westminster.

At Westminster Hospital the Christmas Day celebrations were begun by carols sung by the boys of the hospital choir, after which Father Christmas, in the person of the Senior House Surgeon, handed a present to each patient. Dinner consisted of the usual Christmas fare, and in the afternoon there was a concert in the Board Room for the convalescents, under the direction of the Senior House Physician. A band of pierrots, consisting of members of the staff, gave much amusement, and there was a heavily laden Christmas-tree in the children's ward. The patients in the men's ward were accorded the much-coveted privilege of being allowed to smoke. A special feature at the Westminster is always the bran pies, and this year they were even more original in shape and enormous in size. Each ward possessed one, and there were to be seen a huge snowball, a windmill, a very artistic basket of flowers, a gigantic cracker, a post-office, and a balloon.

### Charing Cross.

The celebrations at Charing Cross Hospital on Christmas Day were of an informal character. The wards were all most tastefully decorated by the sisters, and it was wonderful how much variety and originality were displayed. In one ward pale blue was the prevailing note, and everything harmonised with it; in another it was all white and yellow; while in a third crimson was the predominant shade. Scores of fairy lamps of varied hues produced a charming effect. The patients and their friends had a capital tea in the afternoon, and some pierrots caused great enjoyment. A new departure was made in the evening, when all the nursing staff dined with the resident staff, and afterwards spent a very pleasant evening together. On Boxing Day the hospital was again *en fête*. This day was chiefly for the entertainment of the staff. Nurses past and present, students, members of the medical and surgical staff, and the "honoraries" all trooped into the gaily decorated wards, where a sister was to be found in each, presiding over tea. There was a piano in every ward, and from four o'clock onwards concerts were the order of the day.

Royal Free.

Christmas Day was spent very quietly at the Royal Free Hospital, the wards, for the most part, being busy with heavy and serious cases. Last week the students gave a nigger entertainment one night, and the next evening the wards echoed with carols, which they had been industriously practising for several weeks. The performance showed that the "lady-doctors" are not the somewhat grim blue stockings they may appear to the popular imagination, and there was ample evidence of the sympathy which exists between them and their patients. The wards were prettily decorated with holly, evergreens, and fairy-lamps, the new polished floors reflecting the brightness of the scene. In Boys' Ward stood a fine Christmas-tree, which was visited throughout the day by such patients from elsewhere as could journey so far. But a great feature of Christmas Day homely tea-party each was permitted on her own account. Friends were admitted from two to six, and one or two "stayed to tea." To those who have known the hospitals in the past many changes were apparent. The sisters' quarters in particular are a great improvement, and the flat roof on to which they lead has advantages which only hard-worked nurses can fully appreciate.

Samaritan.

Standing in the heart of one of the most crowded districts of London, the Samaritan Hospital, which has done such good work for so many years, is only just settling down under its new régime. The wards are still small, though in many cases two have been thrown into one, and the nursing is now conducted on a more orthodox system. In these circumstances it was impossible to do much in the way of entertainment, but a home-like air pervaded the place, and smiling faces were met with in every ward. Patients' friends were admitted, apparently for as long as they liked to stay, and amateur performances on the piano gave general satisfaction. The decorations, which were very bright and pretty, had mostly been made by the patients themselves, and deftly fashioned paper chains and ornaments and silk lamp-shades were the admiration of the sisters. In the afternoon the choir boys from Holy Trinity arrived and sang carols, and more than one poor woman's eyes filled with tears as the fresh young voices harmonised in some old familiar strain.

Queen Charlotte's.

At Queen Charlotte's Hospital at this season the visitor finds her welcome on the very threshold, for just inside the great portals hangs suspended a holly-wreathed banner bearing the familiar Christmas greeting. Holly, indeed, was everywhere, and upstairs the wards were transformed into bowers of fresh green foliage. The 25th was, as usual, a busy day, and many little Christmas babies first opened their eyes on the light within its grimy walls. Of course entertainments of any formal character were out of the question. The husbands came to tea, everyone was kind and pleasant, and Matron presented each mother and babe with a bundle of warm clothing. At 9 p.m. a pretty little scene was enacted. The lights—which are incandescent gas—were all lowered, and a long procession of some 80 nurses, clad in their pure-white uniform, went round the hospital, and into every ward, carrying lighted Chinese lanterns and singing Christmas hymns and carols. Truly a fit setting—this house of birth—for such a picture! This ended the day for the patients. But the nurses trooped down to their pretty sitting-room, and sang and danced, just as if they had not been on their feet all day, till midnight tolling from St. Pancras Church frightened them away, like so many Cinderellas, to their beds.

(*To be concluded.*)

### A CHRISTMAS NUMBER WRITTEN BY NURSES.

*** It is intended to make the feature of our Christmas Number of 1906 a series of incidents occurring at the Christmas season this year, and we therefore invite nurses engaged in general or special hospitals, in Poor-law infirmaries, in mental institutions, and in district or private work, whether at home, in the Colonies, or abroad, to send in contributions, varying from 500 to 1,000 words, not later than March 31 next. Original photographs illustrating the articles will be very acceptable, and these and the literary contributions used will, of course, be paid for at the usual rate. Preference will naturally be given to the most carefully and brightly written accounts, but incidents of exceptional interest will not be passed over because the writer is deficient in literary style and composition. The contributions should in all cases be addressed to the Editor of the Nursing Section of The Hospital, and marked outside "Christmas Incidents."

### Our Christmas Distribution.

The following letters have been received by us from the recipients of parcels of garments which we were able, through the kindness of our readers, to distribute :—

The Matron of Guy's Hospital writes : "Please accept our thanks for the gifts you have so kindly sent for our patients. They will be most acceptable."

The Matron of the New Hospital for Women, 144 Euston Road, N.W., writes : "I wish to thank you for the very nice parcel of clothing for the in-patients."

The Secretary of St. Mary's Hospital for Sick Children, Plaistow, E., writes : "Please accept my warmest thanks for your kindly present of clothing and toys, which will be most useful among the in-patients."

The Matron of Evelina Hospital for Sick Children, Southwark, writes : "I beg to acknowledge with many thanks the useful and pretty clothing, and the nice dolls, etc., for the sick children in the hospital."

The Secretary of the East London Hospital for Children, and Dispensary for Women, Shadwell, E., writes : "I am desired by the Board of Management to tender you their best thanks for your very welcome present of clothes and toys, and to assure you that the gift is much appreciated."

The Lady Superintendent of the Mount Vernon Hospital for Consumption and Diseases of the Chest, Hampstead and Northwood, writes from Northwood Hospital, Middlesex : "Please let me thank you very heartily for the generous way in which you have responded to our appeal this morning in sending me such a beautiful parcel of warm useful clothing. Knowing how many calls you have, I think that you have treated us most generously."

The Matron of the Metropolitan Hospital, Kingsland Road, N.E., writes : "Please accept our very grateful thanks for the beautiful garments, etc., which you have sent us for our patients at Christmas; we shall find them so useful, for it is a great undertaking to clothe this large 'family,' and all the things you have sent are so acceptable and good. The women's petticoats are especially appreciated; they are so nice to put on when the patients first get up."

The Matron of West Ham and East London Hospital, E., writes: "It was with very great pleasure and thankfulness that I received your large parcel of useful and warm garments. The distress in this borough of West Ham is very great this winter, and I was finding it very difficult even to get toys for our children, and far more difficult to get any clothing for the adult patients. Please accept my very sincere thanks for your great kindness in sending me such a generous present."

The Lady Superintendent of the East End Mothers' Lying-in Home, 394 and 396 Commercial Road, E., writes: "I beg to acknowledge with most grateful thanks your kind donation of warm garments to our poor in-patients. The want and misery around us is fearful, and it is impossible to exaggerate the thankfulness with which our poor women receive any help we are able to give them. A warm garment for themselves or infants is most highly appreciated, and I am most grateful to the kind donors who so generously send us gifts."

### Everybody's Opinion.

[Correspondence on all subjects is invited, but we cannot in any way be responsible for the opinions expressed by our correspondents. No communication can be entertained if the name and address of the correspondent are not given as a guarantee of good faith, but not necessarily for publication. All correspondents should write on one side of the paper only.]

### CORROSIVE SUBLIMATE AND OPHTHALMIA.

"Unjustly Treated" writes: In the summer I joined a home, and was engaged to nurse a maternity case for one of the local doctors. The confinement was a perfectly normal one, but through some oversight, in consequence of the child being born in a slightly asphyxiated condition, the eyes were not attended to at birth, either by doctor or nurse. About seven hours after birth the child showed symptoms of ophthalmia—namely, redness of eyes, swollen eyelids, and profuse yellow discharge from the eyes. Having had good ophthalmic experience I immediately used boracic acid lotion and corrosive sublimate, and telephoned to the doctor, telling him what was the matter and asking for instructions. He ordered boracic lotion only, but never saw the eyes until 24 hours after birth. I attended to the eyes every half hour or more frequently, so of course they were very much improved upon the doctor's arrival the following morning, and with constant care and attention were perfectly all right in a few days' time. I told the doctor I had thoroughly washed the eyes and used 1-4000 corrosive sublimate. To my intense surprise he immediately told me, and before my patient, that I had caused the trouble by the use of the 1-4000 solution. I might add that I made no vaginal examination, and that the child was thickly covered with Vernix caseosa at birth. The following morning brought a letter from the acting matron of the home, saying: "Dr. —— has told me what you have done to baby's eyes. How could you do such a thing on your own responsibility and without any necessity? You ran the risk of ruining the child's sight, to say nothing of the unnecessary suffering inflicted upon the poor little helpless mite. It is most grievous," etc., and giving me one month's notice. I replied that it was untrue; the child had ophthalmia neonatorum; that I had always seen corrosive sublimate used, and as a midwife had used it in hundreds of cases myself, and had seen some of the best London consultants—for whom I have worked—always use it in their work—strength, 1-2000—and had never seen any ill effects from its use. Of course, I apologised to the doctor for using the solution without first ascertaining his wishes in the matter, and begged him to retract the statement. This he refuses to do, and although he knows that the child had ophthalmia, that the eyes were not wiped at birth, and that such a statement is likely to damage me professionally, to protect his own professional reputation he allows me to take the blame of a trouble I never caused. In 1903 the London Obstetrical Society advised its midwives to use 1-4000 corrosive sublimate for eyes; Dr. Herman advises 1-2000; Dr. Galabin, 1-2000; others use 1-1000 for dirty cases. I myself have used 1-1000. Has any nurse or midwife ever had even a slight catarrh of the eyes with 1-4000 corrosive sublimate solution or in a stronger solution? I should be glad to have some information on this subject.

[It is quite correct treatment to wash out a baby's eyes with 1-4000 corrosive sublimate solution if symptoms of ophthalmia neonatorum arise. The operation is unlikely to do harm, and immediate and energetic measures are always necessary to save the eyesight in such cases. But it is advisable for nurses to inform the medical attendant, if possible, of any alarming symptoms which may arise, and to assume the responsibility of treatment only when medical assistance is not available within a reasonable period of time.—Ed. The Hospital.]

### Appointments.

[No charge is made for announcements under this head, and we are always glad to receive and publish appointments. The information, to insure accuracy, should be sent from the nurses themselves, and we cannot undertake to correct official announcements which may happen to be inaccurate. It is essential that in all cases the school of training should be given.]

Ashton-under-Lyne Union.—Miss Mary Crossland has been appointed charge nurse. She was trained at Ashton-under-Lyne Union.

Hartlepool Infectious Diseases Hospital.—Miss Mary Whitlock has been appointed matron. She was trained at Sheffield Royal Infirmary, and has since been nurse at Hull Fever Hospital, and has done private nursing in Sunderland, Plymouth, and Blackheath.

Lewisham Infirmary.—Miss Annie W. Gooding has been appointed ward sister. She was trained at Poplar Hospital, and has since been on the Blackheath private staff.

Maidenhead Union Infirmary.—Miss Kathleen Wright has been appointed charge nurse. She was trained under the auspices of the Meath Workhouse Nursing Association at Canterbury Hospital.

Queen Victoria Jubilee Institute for Nurses.—Miss Blanche M. Glover has been appointed inspector. She was appointed Queen's Nurse in July, 1894, and has held the post of superintendent of the Lincolnshire Nursing Association since November, 1898.

The Kent and Tavistock Cottage Hospital.—Miss Lucy Bolton has been appointed sister-matron. She was trained at the Evelina Children's Hospital and Guy's Hospital, London, and she has since been charge nurse at Paddington Green Hospital, sister at Salop Infirmary, and sister at Bristol Royal Infirmary.

Throat and Ear Hospital, Brighton.—Miss Edith E. H. Bruce has been appointed staff nurse. She was trained at Glasgow Royal Infirmary.

### A Book and its Story.

### AN OPTIMIST'S NOTE-BOOK.*

Readers who appreciated the rare quality of a former book, "The House of Quiet," will welcome another written somewhat on the same lines by its author, who, it may be remembered, was spoken of as a man of much culture, holding a high official appointment, which in the prime of life he had to relinquish on account of a sudden seizure that left him confronted with the prospect of enforced inaction for the remainder of his days, intensified by intervals of acute suffering. To trace the golden thread of beauty running through homely, simple things, as well as in those of wider range, is the *raison d'être* of the book. "To worship beauty, innocent beauty of thought, of sound, of sight seems to me to be perhaps the most precious thing in the world, and to hold within it a hope which stretches away even beyond the grave. Out of the silence and nothingness we arise; we have our short space of sight—hearing; and then into silence we depart. Sometimes the path is hard and lonely, and we stumble in miry ways; but sometimes our way is through fields and thickets, and the valley is full of sunset light." The composition of this book was a task apparently set for himself by the author, as an antidote against morbid introspection and dissatisfaction with the changed conditions of his life. It is with an almost child-like simplicity that he prefaces the opening chapter with further expressions of its intention, as follows: "So when I have seen or heard something which gives me joy, whether it be a new place; or, what is better still, an old familiar place transfigured by some happy accident of sun or moon into a mystery; or if I have been told of a generous and beautiful deed, or heard even a sad story that has some seat of hope within it; or if I have met a gracious and kindly person; or if I have read a noble book or seen a rare picture or curious flower; or if I have heard a delightful music; or if I have been visited by one of those joyful and tender thoughts that set my feet in the right way, I will try and put it down, God prospering me. For thus I think that I shall be truly interpreting His love for the little souls of men." A golden rule surely, and one within the reach of all, in one degree or another, whether they be in health or sickness, for the cultivation of content and quiet joy. The reflections vary. Sometimes grave, rarely gay, but consistently suggestive, and covering a wide range of subjects. The author has been reading the life of an artist who had been ennobled, and the felicitations of his friends on the honour conferred give rise to the expression by the artist of pleasure at the congratulations, and at the same time surprise that it should be necessary to wait for such an occasion to express appreciation. A day set apart in the year when they could write to friends whom they admired and loved for being *what they were*, would be much more acceptable to the recipients. This craving of a great artist for sincere expressions of personal appreciation gives the author an opportunity for pressing home a lesson too often neglected, for we, as a people, lose much by our tendency to be silent, even from kind words, for fear of appearing insincere. "Of course, such a custom would be clumsily spoilt by foolish persons if it were to become general, as all things are spoilt which become conventional. But the fact remains that the sweet pleasure of praising, of encouraging, of admiring, and telling our admiration, is one that we English people are sparing of; to our own loss and hurt." Then follows an assertion that it would be well to lay to heart, for it is one that strikes few people, perhaps, in dealing with others: "It is just as false to refrain from saying a generous thing for fear of being thought insincere, and what is horribly called "gushing," as it is to say a hard thing for the sake of being thought straightforward. If a hard thing must be said, let us say it with pain and tenderness, but faithfully. And if a pleasant thing can be said, let us say it with joy, and with no less faithfulness." How many rifts within the lute might be arrested if this wise precept was more generally acknowledged. On friendship and springtime and a particular day in spring when, in company with an old friend, the author walks over the flat fenland he loves so well and watches the subtle changes by which, even on a February day, the lover of nature is cheered, he has many good things to say. "The wind was at work brushing away great inky clouds out of the sky. They came sailing up, those great rounded masses of dark vapour—like huge galleons driving to the West, spilling their freight as they came. The air would suddenly be full of tall twisted rain-streaks, and then would come a bright burst of the sun. But a secret change had come in the night; some silent power filled the air with warmth and balm. And to-day when I walked out of the town with an old familiar friend, the spring had come. . . . When the body and mind are fresh and vigorous these outside impressions often lose, I think, their sharp savours . . . But on such soft languorous days as these—of these days of early spring, when the body is unstrung and the bonds and ties that fasten the soul to its prison are loosed and unbound, the spirit, striving to be glad, draws in through the passages of sense these swift impressions of beauty, as a thirsty child drains a cup of spring water on a sun-scorched day, lingering over the limpid freshness of the gliding element." Past an old mill and an untenanted Georgian manor house, following the line of an old Roman road until they came to their goal, the summit of the wold, where from the grassy vantage-ground they commanded a wide outlook over the plain, the two discuss friendship, its claims and relations. "I do not know why, we were speaking of the taking up of old friendships and the comfort and delight of those serene and undisturbed relations which one sometimes establishes with a congenial person, which no lapse of time or lack of communication seems to interrupt—the best kind of friendship. There is no blaming of conditions that may keep the two lives apart; no feverish attempt to keep up the relation, no resentment if the mutual intercourse dies away. And then, perhaps, in the shifting of conditions, our life is again brought near to the life of one's friend and the old easy intercourse is quietly resumed. My companion said that such a relation seemed to him to lie as near the solution of the question of the preservation of identity after death as any other phenomena of life. . . . If the stuff our thoughts are made of were to alter as the materials of our body alter, the continuity of such an emotion would be impossible. Of course it is difficult to see how, divested of the body, our perceptions can continue; but almost the only thing we are really conscious of is our own identity, our sharp separation from the mass of phenomena that are not ourselves. And if an emotion can survive the transmutation of the entire frame, may it not also survive the dissolution of that frame?" "The Thread of Gold" is a book that does not easily lend itself to a notice like the present one. Only in its own pages can its real beauty be found. It is one particularly suited to convalescents, who in its soothing thoughts may find rest and relief for their own weariness by communion with a spirit that has suffered and is strong.

* "The Thread of Gold." By the Author of "The House of Quiet." (John Murray, 8s.)

### Notes and Queries.

**REGULATIONS.**

**The Editor is always willing to answer in this column, without any fee, all reasonable questions, as soon as possible.**

**But the following rules must be carefully observed.**

**1. Every communication must be accompanied by the name and address of the writer.**

**2. The question must always bear upon nursing, directly or indirectly.**

**If an answer is required by letter a fee of half-a-crown must be enclosed with the note containing the inquiry.**

*Male Nursing Association.*

(88) Please let me know of a good male nursing association.—*J. A.*

Write to the Male Nurses' Temperance Co-operation, 10 Thayer Street, Manchester Square, W.

*Epileptic.*

(89) Can you give me the names of any convalescent homes in the Midlands where a girl aged twenty could go for a rest? She is suffering from epileptic fits. Her relations could pay a small amount towards her support.—*E. R.*

It would be difficult to get her admitted into a convalescent home. Why not try some institution for epileptics only? The Liverpool Home for Epileptics, Maghull, near Liverpool, might be suitable. Write to the Hon. Secretary.

*Visiting by the Clergy.*

(90) Please inform me whether it is the rule in the London hospitals and other larger institutions for the Roman Catholic priests, or any of the clergy, to visit at any time they think fit, under any circumstances, and any condition of the patient.—*Cottage Hospital.*

The clergy only visit patients in London hospitals at stated hours, or by special permission of the authorities.

*A Difficulty.*

(91) I was engaged about September 10th to attend on a lady during her confinement, which she expected about January 15th, and I was engaged from January 1st until February 15th, thereby making my engagement of six weeks' duration. About a month after I was engaged the lady wrote to me and said she had altered her plans and was going away, and would not require me, and offered to remunerate me. I replied, and said I had refused a case owing to her engagement. I have never heard from her since. What should I do, and how much should I get for being disappointed? I always received £2 2s. per week.—*Puzzled.*

In the event of a persistent refusal of remuneration, we are afraid that your only remedy is to take legal proceedings. You are entitled to full fees, but it might be worth your while to accept half.

*Plague Nursing in India.*

(92) Kindly inform me where I should apply for particulars of plague nursing in India.—*J. M. M.*

Write to the Under Secretary of State for India, India Office, St. James's Park, S.W.

*Pensions for Nurses.*

(93) I should be glad to know if there is a Pension Fund for Army nurses apart from the Royal National Pension Fund.—*Dickey Sam.*

The Royal National is the only Pension Fund in the Kingdom for nurses; but Army nursing sisters who retire at the age-limit are entitled to a pension.

*Free Training in Monthly Nursing or Midwifery.*

(94) Will you be so good as to tell me if there is any place in London where training in monthly nursing or midwifery is given free?—*Nurse F.*

Write to the Secretary of the Association for Promoting the Training and Supply of Midwives, Dacre House, Dean Farrar Street, Westminster, S.W.

*Royal British Nurses' Association.*

(95) Will you kindly let me know whether I can become a member of the Royal British Nurses' Association? I hold a three years' certificate from a large infirmary, and also the certificate of the Central Midwives Board.—*J. S.*

Yes, you are eligible for membership. Write to the Secretary of the Association, 10 Orchard Street, Baker Street, W.

**Handbooks for Nurses.**

| | Post Free. |
|---|---|
| "A Handbook for Nurses." (Dr. J. K. Watson.) ... | 5s. 4d. |
| "Nurses' Pronouncing Dictionary of Medical Terms" | 2s. 0d. |
| "Art of Massage." (Creighton Hale.) ... ... ... | 6s. 0d. |
| "Surgical Bandaging and Dressings." (Johnson Smith.) ... ... ... ... ... ... ... | 2s. 0d. |
| "Hints on Tropical Fevers." (Sister Pollard.) ... | 1s. 8d. |

Of all booksellers or of The Scientific Press, Limited, 28 & 29 Southampton Street, Strand, London, W.C.

### For Reading to the Sick.

"WHATE'ER MY GOD ORDAINS IS RIGHT."

Whate'er my God ordains is right,
His will is ever just;
Howe'er He order now my cause,
I will be still and trust.
He is my God,
Though dark my road,
He holds me that I shall not fall,
Wherefore to Him I leave it all.

Whate'er my God ordains is right;
My Light, my Life is He,
Who cannot will me aught but good;
I trust Him utterly;
For well I know,
In joy or woe,
We soon shall see as sunlight clear,
How faithful was our Guardian here.

*S. Rodigast.*

We should learn to trust God, even when the hour is darkest. The morning will surely come, and in its light the things that alarm us now will appear in friendly aspect; and in the forms we have dreaded so much, we shall see the benign face of Jesus as he comes to us in love. The ploughings of our hearts are but the preparation for fruitfulness. The black clouds that appear so portentous of evil pass by, leaving only gentle rain, which renews all the life, and changes desert to garden.

In the Canton of Bern, in the Swiss Oberland, a mountain stream rushes in a torrent toward the valley, as if it would carry destruction to the villages below; but, leaping from the sheer precipice of nearly nine hundred feet, it is caught in the clutch of the winds, and sifted down in fine, soft spray, whose benignant showering covers the fields with perpetual green. So sorrow comes, a dashing torrent, threatening to destroy us; but by the breath of God's Spirit it is changed as it falls, and pours its soft, gentle showers upon our hearts, bedewing our withering graces, and leaving rich blessings upon our whole life.

Another reason why many of God's ways seem so strange to us, is because we see them only in their incompleteness. We must wait until they are finished before we can fully understand God's intention in them, or see the beauty that is in his thought. We stand by the sculptor's block when he is busy upon it with mallet and chisel, and to our eye it appears rough, with no lines of beauty; but we see it afterward, when it is unveiled to the world, and it seems almost to breathe, so perfect is the finished statue.

We are all scholars in God's school. The book of providence is written in a language we do not yet understand; but the passing years, with their experiences, bring riper knowledge, and, as we learn more and more, the painful mysteries vanish.—*Dr. J. S. Miller.*

